# The oncogenic role of CCDC178 in intrahepatic cholangiocarcinoma through LGR4-dependent signaling and immune modulation

**DOI:** 10.1016/j.gendis.2025.101910

**Published:** 2025-10-28

**Authors:** Yunlu Jia, Yanli Wang, Yongxia Chen, Dayong Zheng, Wenguang Fu, Xiaochen Zhang, Yunkun Lu, Jian Ruan

**Affiliations:** a Department of Medical Oncology, The First Affiliated Hospital, Zhejiang University School of Medicine, Hangzhou, Zhejiang 310003, China; b Department of General Surgery, Sir Run Run Shaw Hospital, Zhejiang University School of Medicine, Hangzhou, Zhejiang 310000, China; c Department of Pathology, The First Affiliated Hospital, Zhejiang University School of Medicine, Hangzhou, Zhejiang 310003, China; d Department of Surgical Oncology, Sir Run Run Shaw Hospital, Zhejiang University School of Medicine, Hangzhou, Zhejiang 310020, China; e Cancer Center, Integrated Hospital of Traditional Chinese Medicine, Southern Medical University, Guangzhou, Guangdong 510515, China; f Department of Hepatobiliary Surgery, The Affiliated Hospital of Southwest Medical University, Luzhou, Sichuan 646000, China

Intrahepatic cholangiocarcinoma (ICC) is a highly aggressive malignancy with limited therapeutic options and poor prognosis.^1^^,^^2^ Despite recent advances in surgical techniques and systemic therapies, the molecular drivers of ICC progression remain incompletely understood. Coiled-coil domain-containing proteins (CCDCs) are increasingly recognized for their roles in tumor biology. Among them, CCDC178 has emerged as a candidate oncogene in certain cancers, but its role in ICC has not been previously elucidated.^3^ In this study, we identify CCDC178 as a key oncogenic regulator in ICC and reveal its mechanistic function through leucine-rich repeat-containing G protein-coupled receptor 4 (LGR4)-dependent signaling and immune modulation.

Using transcriptomic data from TCGA and GSE107943 cohorts, we found that CCDC178 expression was significantly elevated in ICC tumors compared with adjacent normal tissues (TCGA: *P* = 0.018; GSE107943: *P* = 0.047) (Fig. 1A). This observation was corroborated by analysis of eight matched tumor and adjacent normal tissue pairs from ICC patients, where both quantitative PCR and Western blotting confirmed increased CCDC178 expression in tumors. Additionally, ICC cell lines (HCCC-9810, RBE, HuCCT-1, CCLP-1) displayed higher baseline expression of CCDC178 compared with normal biliary epithelial cells (Fig. 1B; Fig. S1A and S1B), further supporting its relevance in ICC biology. Clinically, high CCDC178 expression was significantly associated with advanced pathological stage (Stage III–IV: 66.6% of cases), tumor diameter > 5 cm (60.1%), and lymph node metastasis (63.3%), indicating a close link to tumor aggressiveness (Fig. 1C; Fig. S1C and S1D). Kaplan–Meier analysis of the OEP001105 dataset demonstrated that patients in the CCDC178-high group had significantly reduced overall survival compared with the low-expression group (hazard ratio = 1.85, 95% confidence interval: 1.18–2.92, *P* = 0.009) (Fig. 1D). A limitation of our study is that the validation of CCDC178 expression relies on the GSE107943 cohort, while the TCGA-CHOL dataset contains only 36 ICC cases, preventing robust survival analysis. Moreover, no additional large-scale ICC cohorts are currently available for independent validation. Future studies in larger, multi-center cohorts will be needed to further confirm the prognostic and therapeutic relevance of CCDC178. This integrated evidence highlights the prognostic potential of CCDC178 as a biomarker, pending validation in larger cohorts.

**Figure 1:**
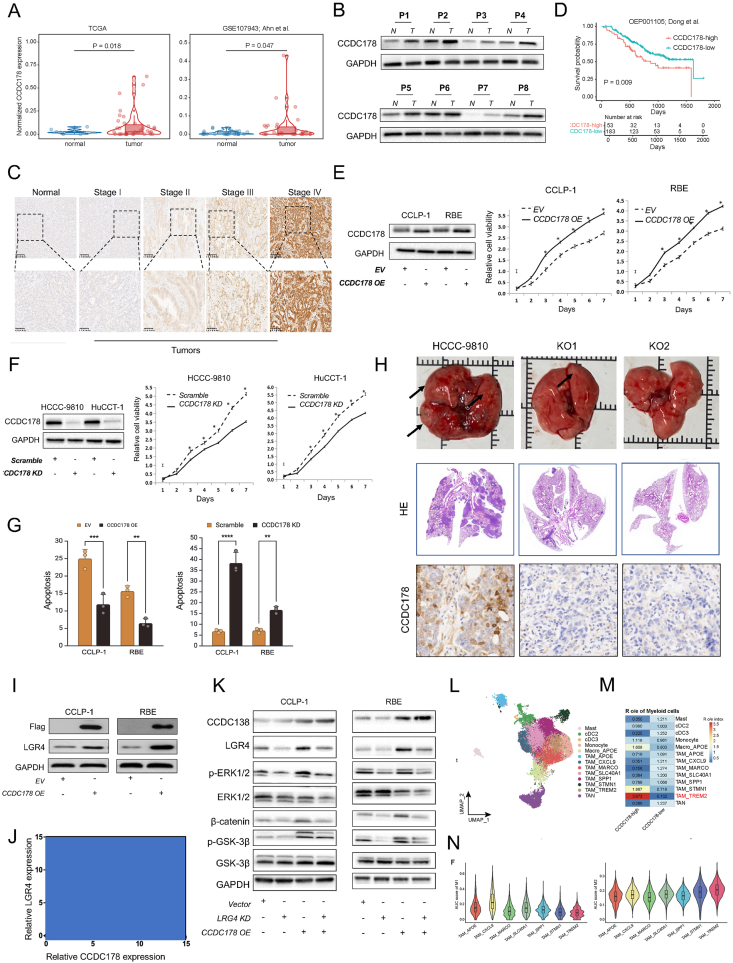
CCDC178 promotes intrahepatic cholangiocarcinoma via LGR4-mediated oncogenic signaling and immune modulation. **(A)** CCDC178 mRNA expression levels were significantly elevated in tumor tissues compared with adjacent normal tissues in the TCGA (*P* = 0.018) and GSE107943 (*P* = 0.047) cohorts. **(B)** Western blotting analyses of paired tumor (T) and adjacent normal (N) tissues from 8 patients with intrahepatic cholangiocarcinoma (P1–P8) revealed elevated protein levels of CCDC178 in tumor tissues. **(C)** Immunohistochemical staining showed a progressive increase in cytoplasmic CCDC178 expression from normal bile duct tissues to stage III–IV tumors. **(D)** Kaplan–Meier survival analysis using the OEP001105 dataset revealed that patients with high CCDC178 expression had significantly worse overall survival than those with low expression (*P* = 0.009). **(E)** Western blotting confirmed successful overexpression of CCDC178 in CCLP-1 and RBE cells, followed by CCK-8 assays, which demonstrated that CCDC178 overexpression significantly enhanced cell proliferation compared with control cells. **(F)** Efficient knockdown of CCDC178 in HCCC-9810 and HuCCT-1 cells was validated by decreased protein and mRNA expression levels. Moreover, silencing CCDC178 resulted in reduced cell proliferation, as indicated by flatter CCK-8 growth curves. **(G)** Flow cytometric analysis of annexin V/PI staining revealed that overexpression of CCDC178 in CCLP-1 and RBE cells significantly reduced apoptotic cell populations compared with the empty vector (EV) control group. Besides, knockdown of CCDC178 markedly increased the percentage of apoptotic cells relative to the scramble control. **(H)** Gross examination of mouse livers showed numerous surface tumor nodules in control groups (HCCC-9810), whereas markedly fewer or no visible lesions were observed in CCDC178-knockout groups (KO1 and KO2). Hematoxylin-eosin staining confirmed extensive tumor infiltration in control livers, which was substantially reduced in the knockout groups. Immunohistochemistry demonstrated strong cytoplasmic CCDC178 expression in control tumors, while KO1/KO2 tumors showed minimal to absent staining, validating effective gene deletion. **(I)** Western blotting analysis showed that CCDC178 overexpression in CCLP-1 and RBE cells increased both Flag-CCDC178 and LGR4 expression. **(J)** Pearson correlation analysis revealed a significant positive correlation between CCDC178 and LGR4 mRNA expression in clinical samples (*r* = 0.61; *P* < 0.0001). **(K)** Western blotting analysis in CCLP-1 and RBE cells showed that CCDC178 overexpression increased phosphorylated ERK1/2 (p-ERK1/2) and β-catenin levels, while LGR4 knockdown reduced their expression, suggesting activation of ERK and Wnt signaling pathways by CCDC178 in an LGR4-dependent manner. **(L)** Subclustering of the myeloid compartment further delineated mast cells, dendritic cells, monocytes, macrophages, tumor-associated macrophages (TAMs), and tumor-associated neutrophils (TANs). **(M)** Ratio of odds enrichment (Ro/e) analysis demonstrated a specific enrichment of TREM2^+^ TAMs in the CCDC178-high group, a population not detected in the CCDC178-low group. **(N)** Functional scoring showed that TREM2^+^ TAMs displayed a predominantly immunosuppressive M2-like phenotype, with higher M2 scores and lower M1 scores.

To further delineate the functional roles of CCDC178 in ICC malignancy, we stratified the OEP001105 dataset based on CCDC178 expression levels into distinct cohorts: CCDC178-high expression group and CCDC178-low expression group (Fig. S1E). KEGG pathway enrichment analysis was performed on the differential genes. The up-regulated genes were related to neuroactivity and metabolism, such as neuroactive ligand–receptor interaction and calcium signaling pathways. The down-regulated genes focused on the activation of glucose metabolism and tumor-associated pathways, including glycolysis and hypoxia-inducible factor 1 (HIF-1) signaling pathways (Fig. S1F and S1G). These findings suggest that metabolic and neuroactive pathways may contribute to ICC aggressiveness, providing a rationale for subsequent mechanistic exploration.

To explore the functional implications of CCDC178, we performed a comprehensive set of *in vitro* and *in vivo* experiments. In CCLP-1 and RBE cells, overexpression of CCDC178 led to significantly accelerated proliferation as measured by CCK-8 assay at 24, 48, and 72 h (Fig. 1E), along with increased migratory and invasive capacities in Transwell assays (Fig. S2A and S2B). In contrast, siRNA-mediated knockdown of CCDC178 in HCCC-9810 and HuCCT-1 cells resulted in markedly reduced proliferation rates and impaired migration and invasion (Fig. 1F; Fig. S2C and S2D). Flow cytometric analysis further revealed that CCDC178 overexpression decreased apoptotic cell populations by 40%–50%, while knockdown increased apoptosis by approximately twofold (Fig. 1G; Fig. S2E and S2F). *In vivo*, orthotopic liver implantation using CCDC178 knockout cells (KO1 and KO2 clones) led to a substantial suppression of tumor growth. Mice in the knockout groups exhibited an average of 2.1 ± 0.6 liver nodules compared with 10.7 ± 1.2 in the control group (*P* < 0.01), with hematoxylin-eosin staining confirming reduced tumor infiltration (Fig. 1H; Fig. S3A and S3B). Immunohistochemistry validated the near-complete absence of CCDC178 expression in knockout tumors. These findings collectively support that CCDC178 plays a critical oncogenic role in ICC by enhancing cell survival and motility *in vitro* and driving aggressive tumor behavior *in vivo*.

Mechanistically, we demonstrated that CCDC178 physically interacted with LGR4, a well-established upstream regulator of both Wnt/β-catenin and extracellular signal-regulated kinase (ERK) signaling cascades.^4^ CCDC178 overexpression resulted in a consistent increase in LGR4 protein levels across multiple ICC cell lines, as confirmed by Western blotting analysis (Fig. 1I). Immunohistochemical staining of ICC tissue sections further revealed strong cytoplasmic co-localization of CCDC178 and LGR4, particularly in high-grade tumors (Fig. S4A). Notably, silencing LGR4 effectively abrogated the enhanced proliferation, migration, and invasion phenotypes conferred by CCDC178, indicating that LGR4 is essential for mediating its oncogenic effects (Fig. S4B–S4D). Western blotting analysis demonstrated that CCDC178 up-regulation induced phosphorylation of ERK1/2 and accumulation of active β-catenin, while LGR4 silencing reversed these changes, confirming the requirement of LGR4 in downstream signaling activation. Luciferase reporter assays demonstrated that ERK and Wnt pathway activities were significantly enhanced in CCDC178-overexpressing cells, whereas these effects were abolished by LGR4 knockdown (Fig. 1K). Consistent results are provided in Figure S5A, supporting the robustness of this observation.

To confirm the functional activity of these pathways, luciferase reporter assays were performed. Overexpression of LGR4 significantly enhanced ERK pathway activity, which was suppressed upon treatment with the ERK inhibitor U0126, confirming that LGR4 is capable of activating ERK signaling (Fig. S5B). Compared with wild-type cells, LGR4-knockout cells showed markedly reduced p-ERK1/2 levels following epidermal growth factor (EGF) stimulation, demonstrating that LGR4 is essential for full ERK pathway activation by EGF (Fig. S5C). Furthermore, co-immunoprecipitation experiments confirmed a direct interaction between endogenous CCDC178 and LGR4 (Fig. S5E), providing mechanistic evidence of a physical and functional CCDC178–LGR4 oncogenic axis that coordinates ERK and Wnt pathway activation in ICC. This further confirms that CCDC178's oncogenic role is dependent on LGR4 signaling.

Given the established role of oncogenic signaling in shaping the tumor immune microenvironment, we next investigated the immune landscape of ICC in relation to CCDC178 expression. To this end, we analyzed a publicly available single-cell RNA-sequencing dataset (GSA: HRA001748) from ICC patients.^5^ Tumor samples were stratified based on CCDC178 expression, and immune cell subsets were profiled using canonical markers (Fig. S6A). Notably, the CCDC178-high group exhibited a marked increase in the proportion of Triggering receptor expressed on myeloid cells 2-positive (TREM2^+^) tumor-associated macrophages (TAMs), with a 3.4-fold enrichment compared with the CCDC178-low group (Fig. S6C). This specific TAM population was absent or minimal in tumors with low CCDC178 expression. TREM2^+^ TAMs are characterized by high expression of apolipoprotein E (APOE), mannose receptor C-type 1 (MRC1 or CD206), and macrophage receptor with collagenous structure (MARCO), markers associated with an immunosuppressive M2-like phenotype. Moreover, M2/M1 polarization scoring showed significantly higher M2 scores and reduced M1 marker expression in TREM2^+^ TAMs of the CCDC178-high tumors (odds ratio = 3.9; 95% confidence interval: 2.1–7.2; *P* < 0.01) (Fig. 1L and M). We hypothesize that LGR4 activation downstream of CCDC178 promotes secretion of lipid mediators and cytokines such as C–C motif chemokine ligand 2 (CCL2) and interleukin-10 (IL-10), fostering a niche that favors macrophage recruitment and M2 polarization.

Our study supports the role of CCDC178 as a potential oncogenic driver, acting through a unique interaction with LGR4. Unlike previously characterized oncogenes in ICC, such as isocitrate dehydrogenase 1/2 (IDH1/2) mutations, fibroblast growth factor receptor 2 (FGFR2) fusions, and Kristen rat sarcoma (KRAS) alterations, which primarily contribute to metabolic reprogramming or canonical signaling activation, CCDC178 exerts dual functions by both activating ERK/Wnt signaling and reshaping the tumor immune microenvironment. Importantly, we show that CCDC178-high tumors are selectively enriched for TREM2^+^ tumor-associated macrophages, which display an immunosuppressive phenotype characterized by elevated APOE, MRC1, and MARCO expression. Although our analysis establishes a strong association between CCDC178 expression and TREM2^+^ TAM enrichment, definitive mechanistic validation using co-culture systems or cytokine secretion assays was beyond the scope of this short-format study. Our findings raise the possibility that targeting the CCDC178–LGR4 signaling axis could attenuate immune suppression and synergize with immune checkpoint inhibitors to enhance therapeutic efficacy in ICC.

Recent reviews on targeted and immunotherapy approaches in ICC emphasize the urgent need for novel biomarkers and rational combination strategies. In this context, our study delineates a dual role for CCDC178 in ICC: it promotes malignant behaviors in tumor cells through LGR4-mediated ERK and Wnt signaling, and it reshapes the tumor microenvironment to favor immune suppression. Targeting the CCDC178–LGR4 axis may therefore represent a promising therapeutic strategy to inhibit tumor progression and enhance anti-tumor immunity in ICC patients.

## CRediT authorship contribution statement

**Yunlu Jia:** Writing – original draft, Resources. **Yanli Wang:** Data curation. **Yongxia Chen:** Resources. **Dayong Zheng:** Resources. **Wenguang Fu:** Writing – review & editing. **Xiaochen Zhang:** Writing – review & editing. **Yunkun Lu:** Visualization. **Jian Ruan:** Supervision.

## Ethics declaration

All animal experiments were approved by the Institutional Animal Care and Use Committee of Zhejiang University, Hangzhou, Zhejiang, China (Approval No. ZJU2023-0186).

## Funding

This study is supported by the “Pioneer” and “Leading Goose” R&D Program of Zhejiang, China (No. 2024C03175), the National Natural Science Foundation of China (No. 82473004, 82000212, 82102814, 81874173, 82100161, 82403160), the Natural Science Foundation of Zhejiang Province, China (No. LMS25H160017, LQN25H160023), the Medical Health Science and Technology Project of Zhejiang Provincial Health Commission of China (No. 2021RC003, 2024KY989), the Zhejiang Provincial Science and Technology Program for Traditional Chinese Medicine (China) (No. 2025ZR142), and the Beijing Xisike Clinical Oncology Research Foundation of China (No. Y-MSDZD2022-0161).

## Conflict of interests

The authors declared no competing interests.

## Figures and Tables

**figs1:**
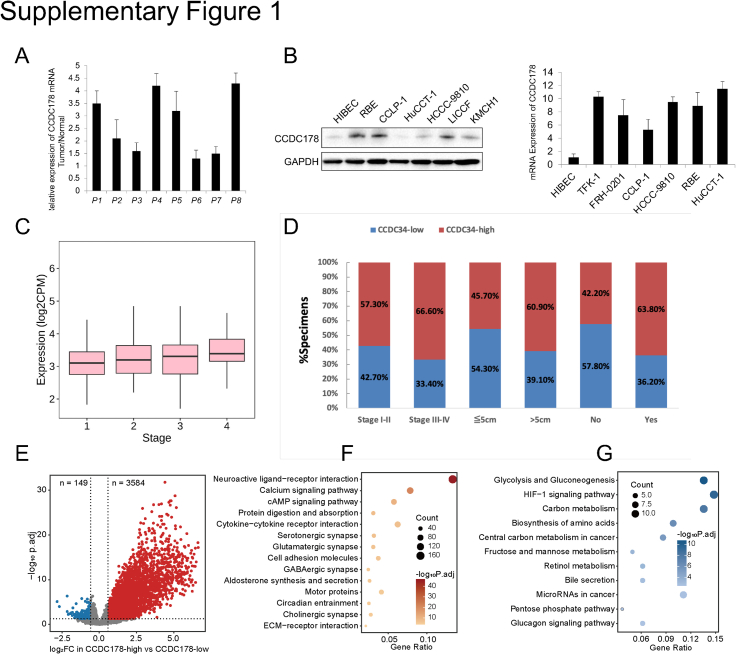


**figs2:**
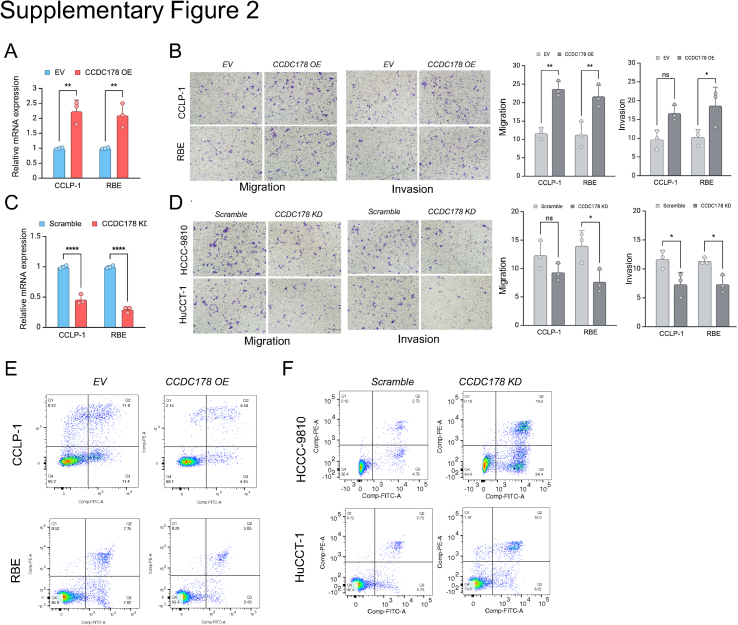


**figs3:**
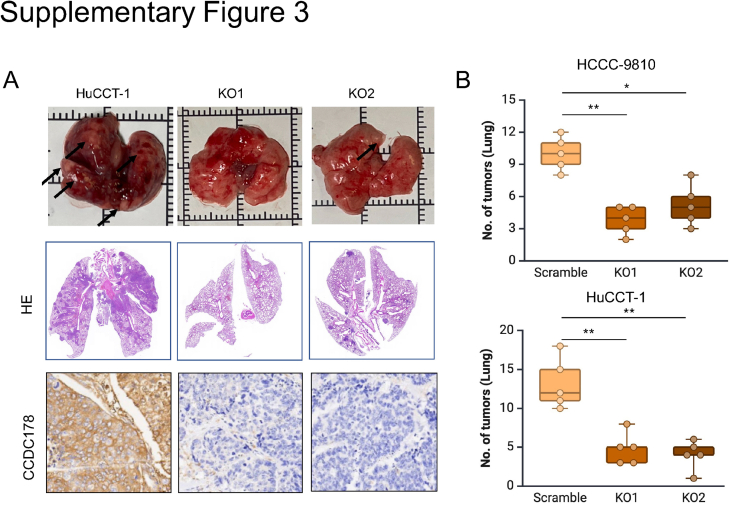


**figs4:**
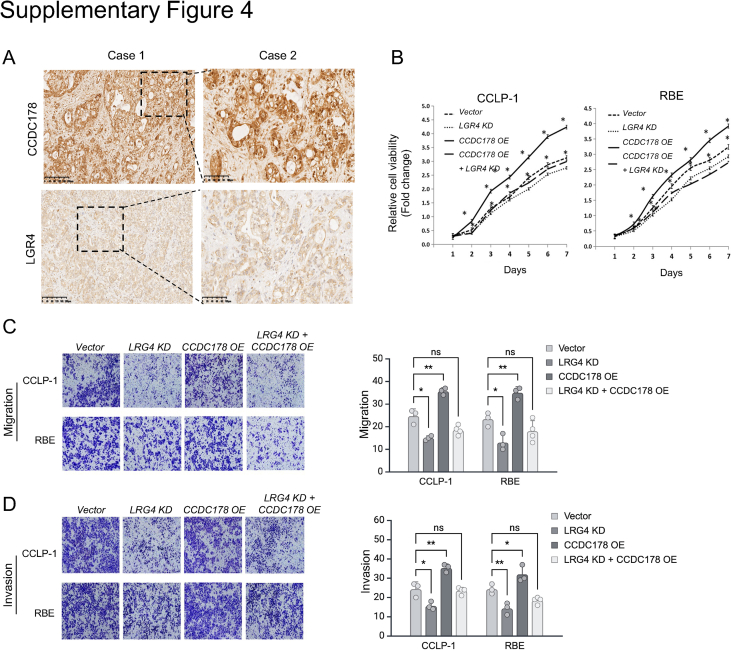


**figs5:**
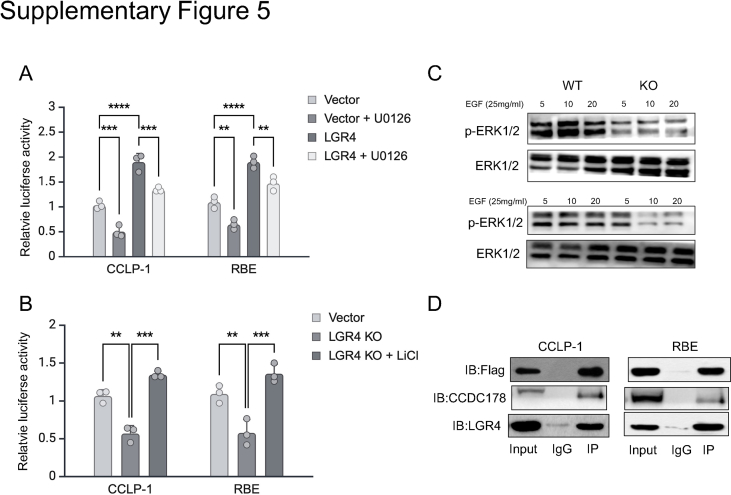


**figs6:**
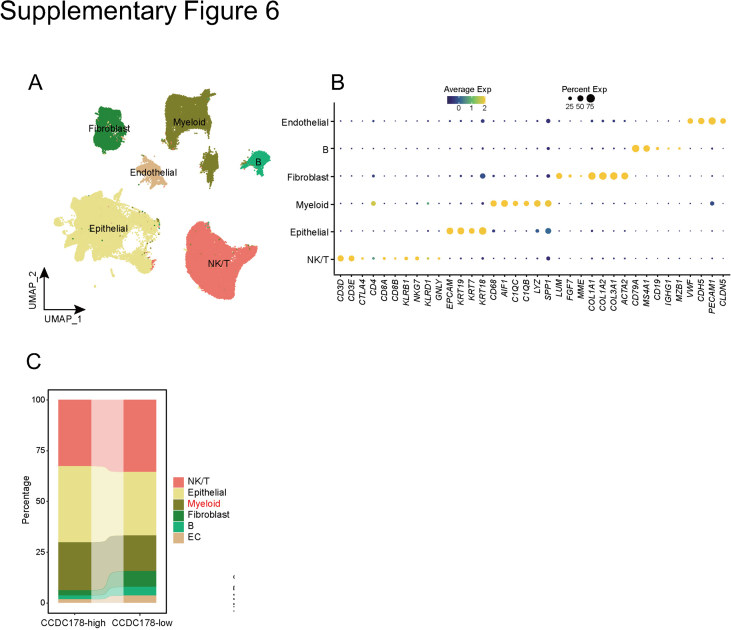


## References

[1] Moris D., Palta M., Kim C., Allen P.J., Morse M.A., Lidsky M.E. (2023). Advances in the treatment of intrahepatic cholangiocarcinoma: an overview of the current and future therapeutic landscape for clinicians. CA Cancer J Clin.

[2] Jia Y., Wan M., Shen Y. (2025). Predictive nomogram integrating radiomics and multi-omics for improved prognosis-model in cholangiocarcinoma. Clin Transl Med.

[3] Hu X., Zhao Y., Wei L. (2017). CCDC178 promotes hepatocellular carcinoma metastasis through modulation of anoikis. Oncogene.

[4] Zhang N., Yuan M., Wang J. (2023). LGR4: a new receptor member in endocrine and metabolic diseases. Endocr Rev.

[5] Alkan F.K., Korkaya H. (2020). Therapeutic utility of immunosuppressive TREM2^+^ macrophages: an important step forward in potentiating the immune checkpoint inhibitors. Signal Transduct Target Ther.

