# Supplementary material for: The oncogenic role of CCDC178 in intrahepatic cholangiocarcinoma through LGR4-dependent signaling and immune modulation
Source: Genes Dis. 2025 Oct 28;13(5):101910. doi: 10.1016/j.gendis.2025.101910 (PMC13101691; doi:10.1016/j.gendis.2025.101910)
Supplement: Multimedia component 1 [file mmc1.docx]

**Materials and Methods**

**Cell Lines and Culture Conditions**

Human intrahepatic cholangiocarcinoma (ICC) cell lines CCLP-1, RBE, HCCC-9810, and HuCCT-1 were obtained from the Chinese Academy of Sciences Committee Type Culture Collection (Shanghai, China) and authenticated by short tandem repeat (STR) profiling. CCLP-1, RBE, HCCC-9810, and HuCCT-1: RPMI-1640 medium (Corning) supplemented with 10% fetal bovine serum (FBS; Gibco) and 1% penicillin-streptomycin (Thermo Fisher Scientific) at 37°C in a humidified atmosphere with 5% CO₂. All cell lines were tested for mycoplasma negativity using the MycoAlert Mycoplasma Detection Kit (Lonza) before experiments and used within 20 passages after thawing.

**Plasmids, siRNAs, and Transfection**

Overexpression plasmids: Full-length human CCDC178 cDNA was cloned into the pcDNA3.1-Flag vector (Invitrogen). LGR4 overexpression plasmid was constructed using the pLVX-IRES-ZsGreen1 vector. Small interfering RNAs (siRNAs) targeting CCDC178 (si-CCDC178#1: 5′-GGAUUAUCCUGAAGAUAAATT-3′; si-CCDC178#2: 5′-CCACAUUGUCUCCUUGAAUTT-3′) and LGR4 (si-LGR4#1: 5′-GGUUGAAGAUCCUCAUUAUTT-3′; si-LGR4#2: 5′-GCAUUGUCCUUGAUUACUUTT-3′) were purchased from RiboBio (Guangzhou, China). Scrambled siRNA was used as a negative control. Cells were transfected with plasmids or siRNAs using Lipofectamine 3000 (Thermo Fisher Scientific) following the manufacturer’s protocol. Transfection efficiency was verified by qPCR and Western blot at 48–72 hours post-transfection.

**Cell Proliferation, Migration, and Invasion Assays**

CCK-8 assay: Cells (1×10³ per well) were seeded in 96-well plates. At 0, 24, 48, and 72 hours, 10 μL CCK-8 reagent (Dojindo) was added, and absorbance at 450 nm was measured. Transwell migration assay: Cells (5×10⁴ per well) were suspended in serum-free medium and seeded into the upper chamber of 8-μm pore Transwell inserts (Corning). The lower chamber contained medium with 10% FBS. After 24 hours, non-migrated cells were removed, and migrated cells were fixed with methanol, stained with 0.5% crystal violet, and counted under a microscope (5 fields/insert).

Invasion assay: Similar to migration assays, but upper chambers were pre-coated with Matrigel (1:8 dilution; BD Biosciences). Cells were incubated for 48 hours before counting.

**Apoptosis Analysis by Flow Cytometry**

Cells were harvested 48 hours after transfection, stained with Annexin V-FITC and propidium iodide (PI) using the Annexin V-FITC Apoptosis Detection Kit (BD Biosciences), and analyzed on a FACSCanto II flow cytometer (BD Biosciences). Data were processed with FlowJo software (v10).

**Orthotopic liver implantation model**

Six-week-old male BALB/c nude mice (Shanghai SLAC Laboratory Animal) were used. CCDC178-knockout (KO1, KO2) or control (HCCC-9810, HuCCT-1) cells (5×10⁶ in 50 μL PBS) were injected into the left liver lobe. Mice were euthanized 4 weeks post-injection. Tumor nodules were counted, and tissues were fixed in 4% paraformaldehyde for H&E and immunohistochemical (IHC) staining. All animal experiments were approved by the Institutional Animal Care and Use Committee of Zhejiang University (Approval No. ZJU2023-0186).

**Immunohistochemistry**

Paraffin-embedded tissue sections (4 μm) were deparaffinized, rehydrated, and subjected to antigen retrieval. Sections were incubated with primary antibodies against CCDC178 (1:200) and LGR4 (1:150) overnight at 4°C, followed by HRP-conjugated secondary antibodies. Staining was visualized with DAB substrate, and sections were counterstained with hematoxylin. Staining intensity was scored by two pathologists blinded to clinical data (0–3: negative to strong).

**Co-Immunoprecipitation (Co-IP)**

Cells were lysed in NP-40 buffer (Beyotime) with protease inhibitors. Lysates were incubated with anti-CCDC178 or anti-LGR4 antibodies overnight at 4°C, followed by Protein G Sepharose beads (GE Healthcare) for 4 hours. Beads were washed, and bound proteins were analyzed by Western blot.

**Luciferase reporter assays**

Cells were co-transfected with ERK or Wnt luciferase reporter plasmids (Addgene) and Renilla luciferase plasmid (for normalization). Luciferase activity was measured using the Dual-Luciferase Reporter Assay System (Promega) 48 hours post-transfection.

**Single-Cell RNA Sequencing Analysis**

The single-cell RNA-seq dataset (GSA: HRA001748) was analyzed using Seurat (v4.0). Cells were clustered based on canonical markers, and subset proportions were compared between CCDC178-high and CCDC178-low groups. Enrichment of TREM2⁺ macrophages was assessed using Ratio of Odds Enrichment (Ro/e) analysis.

**Statistical Analysis**

Data are presented as mean ± SEM from at least three independent experiments. Statistical analyses were performed using GraphPad Prism (v9.0). Differences between groups were analyzed by unpaired two-tailed t-test (two groups) or one-way ANOVA with Tukey’s post-hoc test (multiple groups). Survival curves were generated using the Kaplan–Meier method and compared by the log-rank test. Correlations were analyzed by Pearson’s correlation coefficient. P < 0.05 was considered statistically significant.

**Figure legends.**

**Supplementary Figure 1. High CCDC178 expression is associated with tumor development, poor prognosis, and activation of cancer-related metabolic and signaling pathways**

(A) RT-PCR analyses of paired tumor (T) and adjacent normal (N) tissues from 8 ICC patients (P1–P8) revealed elevated mRNA levels of CCDC178 in tumor tissues.

(B). CCDC178 overexpression was further validated in multiple ICC cell lines at both protein and mRNA levels.

(C) CCDC178 expression levels increased significantly with advancing clinical stage (P = 1.1e-06), based on clinical dataset analysis.

(D) High CCDC178 expression was associated with adverse clinical features, including advanced stage (Stage III–IV), tumor size >5 cm, and lymph node metastasis, with proportions of 66.6%, 60.1%, and 63.3%, respectively. Data are presented as mean ± SEM from at least three independent experiments. *P* < 0.05, **P** < 0.01 were considered statistically significant.

(E) Transcriptomic analysis comparing high and low CCDC178 expression groups (OEP001105) identified 3,584 upregulated and 149 downregulated genes.

(F) KEGG pathway enrichment analysis of the upregulated genes showed significant involvement in neuroactive ligand-receptor interaction and calcium signaling pathways.

(G) Downregulated genes were enriched in pathways related to glucose metabolism and tumor progression.

**Supplementary Figure 2. CCDC178 enhances the malignant phenotypes of ICC cells *in vitro*.**

(A, B) Transwell assays demonstrated increased migratory and invasive capabilities following CCDC178 overexpression.

(C, D) Transwell assays showed that CCDC178 knockdown significantly impaired cell migration and invasion. Data are presented as mean ± SEM from at least three independent experiments. *P* < 0.05, **P** < 0.01 were considered statistically significant.

(E, F) Flow cytometry showed that CCDC178 overexpression reduced apoptosis in CCLP-1 and RBE cells, while its knockdown increased apoptotic cell numbers compared to controls.

**Supplementary Figure 3. CCDC178 knockout inhibits ICC tumor formation and progression *in vivo*.**

(A) Gross liver examination, H&E staining, and immunohistochemistry were performed to assess tumor burden and CCDC178 expression in control and CCDC178-knockout (KO1/KO2) groups.

(B) Quantification of tumor burden revealed a significant reduction in the number of liver tumor nodules per mouse in both KO1 and KO2 groups (n = 5 per group) compared to controls (*P* < 0.05, **P** < 0.01).

**Supplementary Figure 4. CCDC178 promotes malignant behaviors in ICC through an LGR4-dependent mechanism.**

(A) Immunohistochemical staining of ICC tissues demonstrated co-localization of CCDC178 and LGR4 proteins.

(B) CCK-8 assays showed that CCDC178 overexpression enhanced cell proliferation, which was significantly suppressed upon LGR4 knockdown. Cells co-transfected with CCDC178 overexpression and LGR4 knockdown lost the proliferative advantage.

(C, D) Transwell migration and invasion assays indicated that CCDC178 overexpression increased cell motility and invasiveness, while co-knockdown of LGR4 significantly reversed these effects.

**Supplementary Figure 5. CCDC178 activates ERK and Wnt pathways through interaction with LGR4.**

(A) ERK luciferase reporter assays showed that LGR4 overexpression enhanced ERK signaling activity, which was significantly suppressed by the ERK inhibitor U0126.

(B) EGF stimulation induced robust ERK activation in wild-type cells but failed to do so in LGR4-knockout cells, indicating that LGR4 is necessary for full EGF-induced ERK activation.

(C) Wnt signaling activity was reduced in LGR4-knockout cells and rescued upon treatment with the Wnt pathway activator LiCl , confirming LGR4's role in Wnt pathway regulation.

(D) Western blot validation confirmed effective LGR4 knockdown in the corresponding assays. Data are presented as mean ± SEM from at least three independent experiments. *P* < 0.05, **P** < 0.01 were considered statistically significant.

**Supplementary Figure 6. Single-cell transcriptomic analysis reveals that CCDC178 promotes tumor immune evasion by regulating TREM2⁺ macrophages.**

(A, B) Cell-type annotation of ICC tumor microenvironment based on single-cell RNA-seq data (GSA: HRA001748), identifying six major cell populations: malignant epithelial cells, endothelial cells, NK/T cells, B cells, fibroblasts, and myeloid cells.

(C) Proportional analysis of cell types revealed increased abundance of malignant epithelial cells and myeloid cells in tumors with high CCDC178 expression.
